# A Perspective on Accelerated Aging Caused by the Genetic Deficiency of the Metabolic Protein, OPA1

**DOI:** 10.3389/fneur.2021.641259

**Published:** 2021-04-13

**Authors:** Irina Erchova, Shanshan Sun, Marcela Votruba

**Affiliations:** ^1^Mitochondria and Vision Lab, School of Optometry and Vision Sciences, Cardiff University, Cardiff, United Kingdom; ^2^Cardiff Eye Unit, University Hospital of Wales, Cardiff, United Kingdom

**Keywords:** mitochondria, OPA1, mitochondrial dynamics, aging, cellular adaptation

## Abstract

Autosomal Dominant Optic Atrophy (ADOA) is an ophthalmological condition associated primarily with mutations in the *OPA1* gene. It has variable onset, sometimes juvenile, but in other patients, the disease does not manifest until adult middle age despite the presence of a pathological mutation. Thus, individuals carrying mutations are considered healthy before the onset of clinical symptoms. Our research, nonetheless, indicates that on the cellular level pathology is evident from birth and mutant cells are different from controls. We argue that the adaptation and early recruitment of cytoprotective responses allows normal development and functioning but leads to an exhaustion of cellular reserves, leading to premature cellular aging, especially in neurons and skeletal muscle cells. The appearance of clinical symptoms, thus, indicates the overwhelming of natural cellular defenses and break-down of native protective mechanisms.

## Introduction

Autosomal Dominant Optic Atrophy (ADOA), is a progressive ophthalmological condition caused by degeneration of retinal ganglion cells (RGCs) that leads to visual loss (1). It is associated predominantly with mutations in the *OPA1* gene and has variable onset and severity. The pathophysiology of OPA1-related ADOA is believed to be mainly due to haploinsufficiency in OPA1, due to the preponderance of OPA1 mutations that lead to premature translation termination and null mutations (2). This leads to a ~50% reduction in OPA1 protein in most tissues tested (3). In addition, some mutations lead to unstable OPA1 transcripts, due to a premature stop codon, and these appear to be degraded by non-sense mediated mRNA decay, and also lead to haploinsufficiency (4). Thesecond mechanism of disease is linked to missense mutations in the GTPase domain of OPA1, where a dominant-negative effect is postulated to lead to severe “plus” forms of the disease (5).

Affected individuals are usually identified early, as juveniles or adolescents. However, clinical symptoms may appear later in some individuals, such as loss of visual acuity and deficits in color vision (6). Currently, based on the fact that before the onset of symptoms individuals frequently have normal development and good health, supportive treatments are not instituted before the appearance of the clinical symptoms, and disease onset is thus considered age-dependent. As a part of the aging process, the amount of OPA1 protein is believed to decrease, potentially contributing to age-related deterioration of vision, muscle, and memory (7, 8). Moreover, the levels of proteins governing mitochondrial dynamics, which include OPA1, have been found to be significantly altered in Alzheimer Disease (AD) mice and patients (9). On the other hand, there is a mounting body of evidence that suggests that on the cellular level *OPA1* mutations cause abnormalities demonstrable from birth, though many are successfully ameliorated (or compensated for) by cellular adaptive mechanisms. In turn, cellular adaptation, though beneficial as it allows normal development, exhausts the antioxidant system, reduces the control of inflammation, and the supply of adult stem cells, thus depleting natural defenses and therefore potentially accelerates the aging process.

### Severe Developmental Pathologies Are Associated With Homozygous and Heterozygous Mutation of OPA1

OPA1 protein is part of the cellular control of cellular energy production and distribution and thus is essential for development, especially in neurons with long neurites (10). All fetuses carrying homozygous mutations in the *Opa1* gene in murine models die during embryonic development (11, 12). As a result, systematic evidence of homozygous pathology in mammals is rare (13). It has however been possible to investigate cellular changes using artificially created mosaics of homozygous cells in non-mammalian experimental models like *Drosophila* (14, 15), and stress adaptation and life-span changes using nematode, *C. elegans* (16). In humans, homozygous *OPA1* mutations are rarely seen due to presumed fetal loss and when they do occur are associated with very severe developmental conditions, such as encephalomyopathy, muscle weakness, cardiomyopathy, hypertonia, sensory deficits, and more general failure to thrive leading to early death (17). Severe developmental delays and early-onset optic atrophy are also typical for heterozygous mutations causing Behr syndrome, accompanied by spinocerebellar degeneration, ataxia, and sensory deficits (18–21).

### Cellular Deficits With Impaired Mitochondrial Fusion

**Figure 1:**
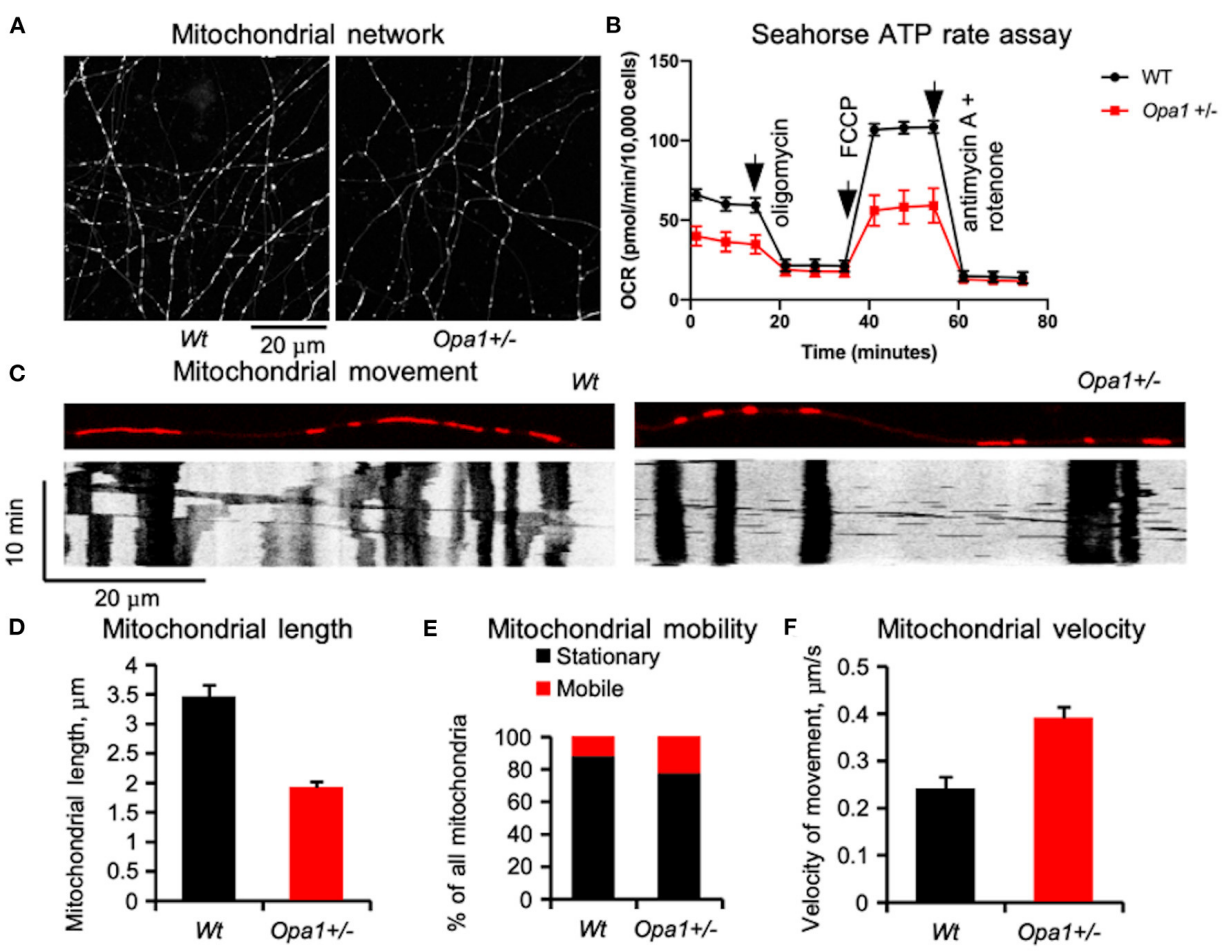
Mitochondrial network in primary retinal ganglion cells (RGCs) in mutant mice (B6;C3-*Opa1*^*Q*285*STOP*^, *Opa1*^+/−^) [adapted from Sun et al. (27)]. **(A)** Tubular mitochondrial network in control and mutant mice labeled in live culture by MitoTracker®Red and recorded by time-lapse imaging on the 8th day *in vitro*. **(B)** Mitochondrial network functionality assessed by Seahorse assay. **(C)** Mitochondrial movement in RGC dendrites is illustrated by automatically generated kymographs using FIJI software. **(D)** Average length of a single mitochondrial segment. **(E)** Distribution of the mitochondrion population classified as stationary and motile. **(F)** Velocities of mitochondrial movement. The increased mitochondrial mobility and velocity may be a compensatory mechanism for a poor cellular distribution network. The data were obtained from 1280 mitochondria in 91 processes of wild-type RGCs and 1205 mitochondria in 92 processes of Opa1+/- RGCs.

Cellular deficits caused by faulty mitochondrial fusion are well-documented. In budding yeast, the tubular mitochondrial network breaks into small spherical segments (22, 23). In *Drosophila*, a similar process affects the motility of the sperm cells and results in male sterility (17). Similar fragmentation is documented in primary cultures of various animal cells (24, 25) and patient-derived induced pluripotent stem (iPS) cells (26), as well as murine retinal ganglion cells from the B6;C3-*Opa1*^*Q*285*STOP*^ mouse, *Opa1*^+/^, (Figure 1) in which there is also accelerated mitochondrial movement (27). Defects in Opa1 primarily affect mitochondrial fusion and motility (28).

### Recruitment of Antioxidant and Inflammatory Defenses

A reduction in mitochondrial quality control, and accelerated mitochondrial movement, are not the only compromises that allow survival. In *Drosophila*, mitochondrial fusion and fission imbalance is tolerable in young flies that mobilize natural antioxidant protection via Nrf2 and Foxo to up-regulate cytoprotective mechanisms (29). It nonetheless leads to accelerated aging and a much shorter life span (29).

Inflammation is another process that affects cell viability. Mitochondrial fusion and OPA1 protein are directly involved in this process via the TNFα-NF-kB–OPA1 regulatory pathway (29, 30). This pathway works via increasing mitochondrial fusion and improving respiratory chain efficiency in response to cellular stress. TNFα (Tumor Necrosis Factor alpha) is a protein (an inflammatory cytokine) produced by macrophages during acute inflammation. It is a signaling molecule that is passed on to other cells. NF-κB (nuclear factor kappa-light-chain-enhancer of activated B cells) is a cytosol-based protein complex that controls transcription of DNA and is involved in cellular responses to stress. While in an inactivated state, NF-κB is in complex with an inhibitory protein, but once activated, it is released from the complex and translocated into the nucleus, where it binds to response elements (specific sequences) of DNA, recruiting co-activators and RNA polymerase. The mRNAs are then translated into proteins changing cell function. Normally, inflammatory stress increases TNFα, which activates NF-kB, and ultimately increases the production of OPA1 protein. The latter part of the pathway NF-kB–OPA1 is also involved in synaptic development (30). The inflammatory response is usually regulated via complex multi-gene signaling adjusting the homeostatic balance of organelles in response to cellular stress (31).

### Embryonic and Adult Stem Cell Recruitment and Depletion

Mitochondrial fusion is essential for normal embryonic development (32). Recently, it has been shown that in human stem cells OPA1 haploinsufficiency causes impairment in neural stem cell self-renewal, thus causing age-dependent depletion, leading to reduced adult neurogenesis and cognitive deficits (33). Similarly, depletion of Opa1 protein affects both stem cell identity and self-renewal, causing age-dependent depletion of adult stem cells and thus possible deficits in adult neurogenesis in a mouse model with a mutation in Dynamin-related Protein gene (DRP) (34). Several recent studies have looked into the molecular mechanisms of this depletion. Sênos Demarco et al. showed that in *Drosophila* stem cells, depletion of Opa1 leads to activation of Target of Rapamycin (TOR) and a marked accumulation of lipid droplets, thus reducing the capacity of stem cells for self-renewal (35). In *Drosophila*, Sandoval et al. showed that mitochondrial fusion regulates larval growth and synaptic development *via* steroid hormone production (36). Moreover, in genetically modified human embryonic and patient-derived induced pluripotent stem cells OPA1 haploinsufficiency leads to aberrant nuclear DNA methylation and thus alters DNA transcription in neural progenitor cells. For instance, the transcription factor needed for GABAergic neuronal development is suppressed, causing reduced generation of GABAergic interneurons, whereas the formation of glutamatergic neurons is not affected (33). The potential reduction in the generation of GABAergic interneurons requires further investigation and exploration, but theoretically, this could result in a more vulnerable neural network, which might require more repair or maintenance. A recent example of the effect of a poorly developed GABA-ergic network has recently been discussed in optic nerve hypoplasia and autism. Clinical observations and recent reports indicate a high frequency of autism spectrum disorders (ASD) in children with optic nerve hypoplasia (ONH) (37, 38). In children with ONH, there are additional characteristics of ASD beyond those attributable to visual impairment alone, such as echolalia and stereotypic motor movements.

### Cellular Mechanisms of Accelerated Aging in Individuals With OPA1 Mutation

In *Drosophila*, experimental oxidative stress was seen in mitochondrial areas abnormally rich in myelin without cytochrome oxidase activity (39). Oxidative stress and up-regulated production of reactive oxygen species (ROS), mostly by mitochondria, are the main factors causing tissue damage. Though these processes are likely to occur independently of mutation, the early deployment of antioxidant defenses leaves limited capacity to cope with additional stress caused by mitochondrial aging. If tissue damage triggers an inflammatory response, then there is also a potentially limited ability to mobilize cellular resources or suppress inflammation. Reduced mitochondrial quality control, adopted early on to prevent degradation of functional, but fragmented mitochondria, also contribute to accelerated aging, by failing to identify and renew damaged mitochondrial fragments in a timely fashion. It is involved in the early loss of skeletal muscle mass and strength in OPA1 mutation carriers (40). Casuso and Huertas recently reviewed the topic in light of the support and protection offered by regular physical exercise (41). The down-regulation of mitochondrial fusion is linked with age-dependent axonal degeneration, with the visible accumulation of fragmented mitochondria in the axons without mitophagy (42).

**Figure 2:**
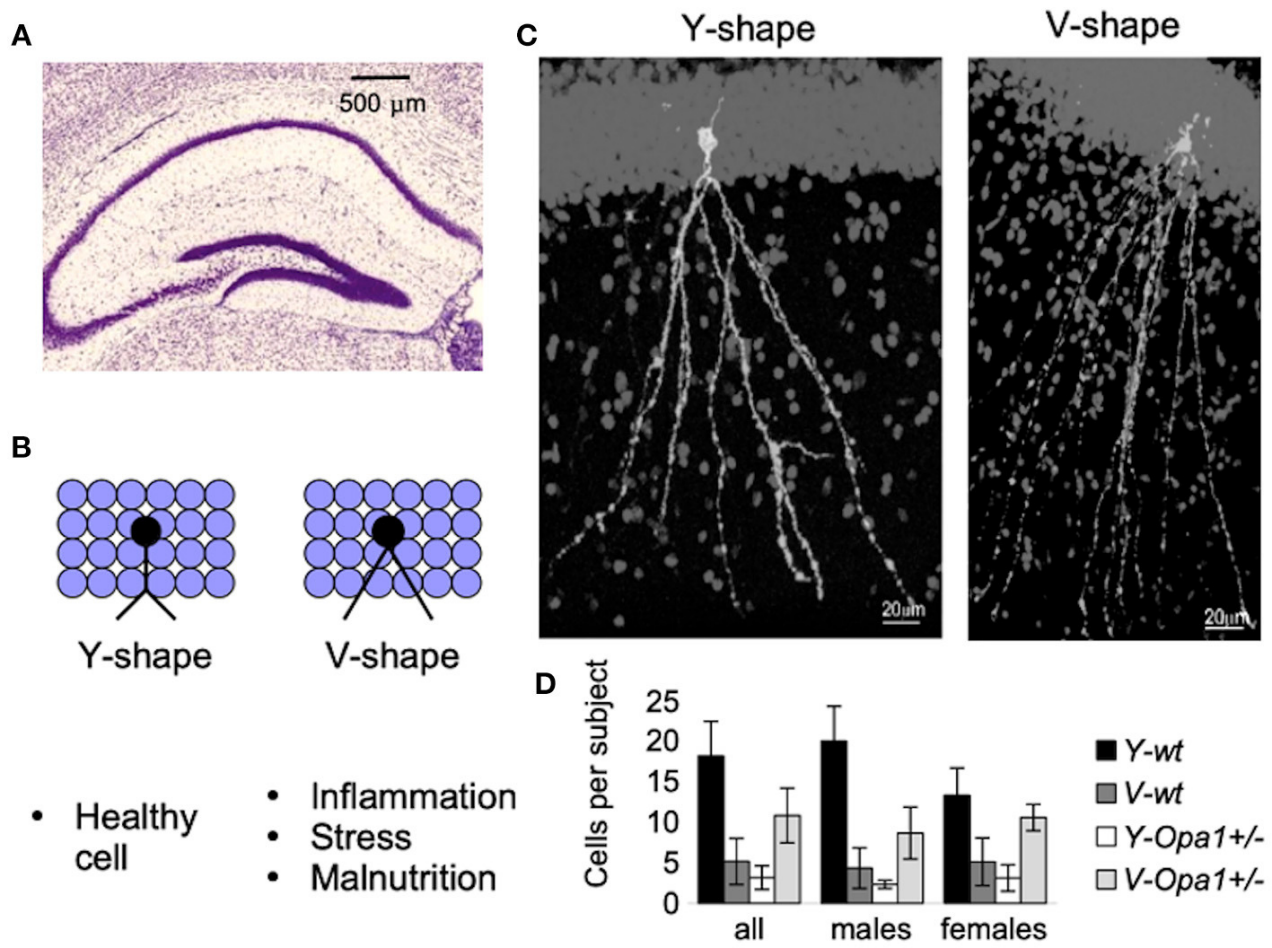
New-born neurons are continuously being added to the hippocampal Dentate Gyrus (DG) throughout adulthood **(A)**. Detrimental factors (such as schizophrenia, stress, Alzheimer's disease, seizures, stroke, inflammation, dietary deficiencies, or the consumption of drugs of abuse or toxic substances) and neuroprotective factors (physical exercise and environmental enrichment) influence maturation and morphology of new-born granular cells [reviewed in Llorens-Martín et al. (45)]. For example, there is a striking difference in the shape of the apical dendrites **(B)**. These morphological alterations may have important consequences (such as change in the pattern of synaptic connections, or change in the pattern of dendritic summation, and ultimately, change in cell excitability and functionality). An example of granular cells with different dendritic shafts are represented in **(C)**. Though both wt and Opa1 +/- animals had both types of granular cell morphology, in aged mice, the Y-type was attributed to 80% of all Diolistically labeled DG granular cells in wt [see Bevan et al. (44) for the details]. By contrast, in age-matched Opa1 +/- animals more cells were of V-type **(D)**. These changes are likely to be induced by a combination of stress factors in conjunction with impaired adult hippocampal neurogenesis.

Apart from the physical symptoms of aging associated with failing body strength and health, there is cognitive aging, characterized by increased anxiety and reduced working memory. On a cellular level, the symptoms are associated with synaptic loss in pyramidal cells and reduced numbers of inhibitory cells (especially somatostatin neurons) involved in the signaling pathways (43). We recently characterized subtly reduced working memory in *Opa1* mutant mouse (B6;C3-*Opa1*^*Q*285*STOP*^, *Opa1*^+/−^) (44). We found that hippocampal CA1 cells showed age-related loss of synapses, developmental abnormalities such as the reduction in the number of GABA-ergic neurons, and defects in adult neurogenesis, which would contribute to the observed effect. Figure 2 illustrates our additional finding in the Dentate Gyrus (DG) region of aged mutant mice. The changes were similar to those observed by Llorens-Martín et al. and are decremental (45).

## Discussion

Recent studies highlight the fact that fusion/fission mitochondrial dynamics and cellular metabolism are coupled: in cultured cell lines, elongated mitochondria are observed in conditions associated with increased ATP requirements (46, 47), whilstin nutrient-restricted conditions, mitochondria form large interconnected networks (48). When fusion is reduced secondary to nutrient excess or genetic abnormality (such as OPA1 deficiency) mitochondrial fragmentation promotes mitochondrial degradation through mitophagy (49). It appears that elongated shape and a fused mitochondrial network protect individual mitochondria from autophagosomal degradation (50, 51). When the tubular network is fragmented not only does energy production decrease but mitochondria themselves are at risk of degradation. To protect mitochondria when the tubular network is compromised, the cellular mechanism responsible for “quality control” is adjusted (52), allowing a larger quantity of disconnected mitochondria to circulate in the cell. This adjustment helps to maintain a sufficient number of fragmented mitochondria to meet the immediate cellular demands in development when most mitochondria are healthy, but with time might lead to accumulation of damaged mitochondria. At present, it is not clear whether mitochondrial fragmentation itself causes any deficit in cellular function, although the mitochondrial network does influence cellular functioning. In cultured HeLa cells, for example, the mitochondrial network interacts with the endoplasmic reticulum (ER) and uses Ca^2+^ signals to rapidly tune ATP production to the demands of the cell (53). However, in the rat cardiac muscle, the developmentally fragmented mitochondrial network later assumes a tubular structure (54). In post-natal primary RGC culture from the B6;C3-*Opa1*^*Q*285*STOP*^, *Opa1*^+/−^, mouse the mitochondrial network forms tubular structures but remains, nonetheless, more fragmented compared to controls, and levels of ATP production are reduced (Figure 1). Another important function of the mitochondrial network is the rapid spatial distribution of energy resources to distant cellular compartments. This function may be partially compensated via increased mitochondrial movement (27).

Moreover, a poorly controlled exuberant inflammatory response leads to tissue deterioration and accelerated aging. The chronic inflammatory response in cell lines and skeletal muscle caused by OPA1 deficiency is well-documented in patients and animal models (55–57). In addition, the adequate response to sepsis caused by pulmonary infection, whichoften complicates other chronic conditions, also requires rapid up-regulation of OPA1 (58). In post-mortem analyses of lung tissue obtained from areas with mild and severe emphysema, impaired fusion characterized by a low quantity of OPA1 protein is correlated with disease severity (59). Evidence from our lab suggests that levels of signaling protein NF-kB are already elevated in healthy Opa1 +/- mice compared to controls without changes in the levels of TNFα. This suggests that developmental adaptive mechanisms are likely to recruit a part of the inactive inflammatory pathway to alleviate cellular deficits in OPA1 during synaptic development (60–62). Unfortunately, this early recruitment of the regulatory pathway may compromise normal inflammatory responses, resulting in chronic inflammation or poor outcome in the case of severe acute inflammation (63, 64).

The aging process in mitochondrial networks at the microscopic level is very different from the tubular fragmentation described above. It characterized by mitochondrial swelling, reduced cristae, and damaged membranes (65, 66). Accelerated aging does not only manifest itself in sensory and cognitive deficits. There are numerous subtle changes that do not manifest themselves in everyday life. For example, recent studies showed that there is an elevated risk of cardiovascular conditions and reduced capacity for a successful recovery. By using C.elegans, Machiela et al. showed that disrupted mitochondrial fusion changed the normal pattern of responses to cellular stress. Cells became more resistant to both heat and oxidative stress, but more sensitive to osmotic variations and hypoxia. Sensitivity to hypoxia is critical in recovery from ischaemic stroke (67). Guo et al. showed that the increased risk of cerebral vascular injury in diabetic patients is partially due to chronically reduced levels of OPA1. They also reported more severe damage in this group of patients (68). Accordingly, Lai et al. demonstrated that rapid restoration of OPA1 levels after stroke reduces neuronal death and improved both survival and recovery of functions (69). Similarly, Xin and Lu showed in a murine model, that *Opa1* expression was down-regulated in infarcted hearts, but *Opa1* overexpression protected cardiomyocytes (70). Simulated ischaemia in the cardiac myogenic cell line H9c2 cells reduced OPA1 protein levels resulting in mitochondria fragmentation and apoptosis (71).

Thus, in this “Perspective” we summarize the evidence that *OPA1* haploinsufficiency affects cellular functions from the molecular perspective of natural cellular resistance during development and adulthood. Deficits in OPA1 protein impact mitochondrial fusion, reduce cellular energy supply and thus impair cell survival. From the clinical perspective, this means that patients, identified as having a pathological mutation, may benefit from being monitoredbefore, or in the absence of, any clinical symptoms of disease. This could include careful multi-modal imaging of the retina and optic nerve and functional investigation with electrodiagnostic tests. Pre-symptomatic screening would contribute valuable clinical information allowing for the identification of markers of early disease and putative biomarkers that would be essential in the testing of novel therapeutic interventions. It also adds some weight to the idea that by supporting natural defenses, such as maintaining a healthy diet, avoiding smoking and alcohol consumption, and a regular exercise regime throughout the normal lifespan, it may be feasible to delay the onset of premature aging. Smoking is known to disturb mitochondrial function, and may thus be a factor that helps accelerate the onset and progression of visual loss in patients with mutations that impair mitochondrial function [as for example, in Leber Hereditary Optic Neuropathy and ADOA (72)].

There are many further potentially important research questions, such as why and how mitochondria in different tissues differ and whether this affects the apparent different rates of aging in different body tissues, which we would suggest may be worth addressing in future research.

## Data Availability Statement

The raw data supporting the conclusions of this article will be made available by the authors, without undue reservation.

## Ethics Statement

The animal study was reviewed and approved by UK PPL Home Office PP7147250.

## Author Contributions

IE: conceptualization, methodology, investigation, writing-original draft preparation, and visualization. SS: investigation, visualization, writing-reviewing, and editing. MV: resources, writing-reviewing and editing, supervision, and funding acquisition. All authors: contributed to the article and approved the submitted version.

## Conflict of Interest

The authors declare that the research was conducted in the absence of any commercial or financial relationships that could be construed as a potential conflict of interest.
